# Real-world effectiveness of GLP-1 receptor agonist-based treatment strategies on “time in range” in patients with type 2 diabetes

**DOI:** 10.3389/fphar.2024.1370594

**Published:** 2024-03-07

**Authors:** Yongru Chen, Jingxian Chen, Shuo Zhang, Dan Zhu, Feiying Deng, Rui Zuo, Yufei Hu, Yue Zhao, Yale Duan, Benwei Lin, Fengwu Chen, Yun Liang, Jiaxiong Zheng, Barkat Ali Khan, Kaijian Hou

**Affiliations:** ^1^ The First Affiliated Hospital of Shantou University Medical College, Shantou, China; ^2^ School of Public Health, Shantou University, Shantou, China; ^3^ Shantou University Medical College, Shantou, China; ^4^ Department of Endocrine and Metabolic Diseases, Longhu People’s Hospital, Shantou, China; ^5^ School of Medicine, Tulane University, New Orleans, LA, United States; ^6^ Department of Medical Affairs, Hanson (Shanghai) Health Technology Co, Ltd, Shanghai, China; ^7^ School of Physiology, Pharmacology and Neuroscience, University of Bristol, Bristol, United Kingdom; ^8^ Drug Delivery and Cosmetic Lab (DDCL), Gomal Center of Pharmaceutical Sciences, Faculty of Pharmacy, Gomal University, Dera Ismail Khan, Pakistan

**Keywords:** type 2 diabetes, time in range, glucagon-like peptide-1 receptor agonists, continuous glucose monitoring, oral antidiabetic drugs

## Abstract

**Background:** Diabetes affects millions of people worldwide annually, and several methods, including medications, are used for its management; glucagon-like peptide-1 receptor agonists (GLP-1RAs) are one such class of medications. The efficacy and safety of GLP-1RAs in treating type 2 diabetes mellitus (T2DM) have been assessed and have been shown to significantly improve time in range (TIR) in several clinical trials. However, presently, there is a lack of real-world evidence on the efficacy of GLP-1RAs in improving TIR. To address this, we investigated the effect of GLP-1RA-based treatment strategies on TIR among patients with T2DM in real-world clinical practice.

**Methods:** This multicenter, retrospective, real-world study included patients with T2DM who had previously used a continuous glucose monitoring (CGM) system and received treatment with GLP-1RAs or oral antidiabetic drugs (OADs). Patients who received OADs served as controls and were matched in a 1:1 ratio to their GLP-1RA counterparts by propensity score matching. The primary endpoint was the TIR after 3–6 months of treatment.

**Results:** According to propensity score matching, 202 patients were equally divided between the GLP-1RA and OAD groups. After 3–6 months of treatment, the TIR values for the GLP-1RA and OAD groups were 76.0% and 65.7%, respectively (*p* < 0.001). The GLP-1RA group displayed significantly lower time above range (TAR) and mean glucose values than the OAD group (*p* < 0.001). Subgroup analysis revealed that, compared with the administration of liraglutide, the administration of semaglutide and polyethylene glycol loxenatide (PEG-Loxe) significantly improved TIR over 3–6 months of treatment (*p* < 0.05).

**Conclusion:** These real-world findings indicate that GLP-1RA-based treatment strategies could be superior to oral treatment strategies for improving TIR among patients with T2DM and that once-weekly GLP-1RA may be more effective than a once-daily GLP-1RA.

**Clinical trial registration:** http://www.chinadrugtrials.org.cn/index.html, identifier number ChiCTR2300073697.

## 1 Introduction

Diabetes is a chronic metabolic disorder affecting millions of people worldwide, and its prevalence is increasing. Specifically, the International Diabetes Federation (IDF) estimated that worldwide, 537 million adults aged 20–79 lived with diabetes in 2021 (Sun et al., 2022). China has the largest population with diabetes in the world in 2021, having reported that the number of patients with diabetes was 140.9 million, accounting for 26.2% of the total reported cases globally (Sun et al., 2022). Chronic hyperglycemia often occurs in those with diabetes and can result in various tissue damages, particularly chronic injuries and functional impairments in the cardiovascular, renal, ocular, and neurological systems (Banday et al., 2020).

The continuous glucose monitoring (CGM) system is a minimally invasive system that monitors blood glucose levels by detecting the glucose concentration in subcutaneous interstitial fluid, providing round-the-clock blood glucose information (Cappon et al., 2019). Time in range (TIR) is a new blood glucose index different from fasting blood glucose, postprandial blood glucose, glycated hemoglobin (HbA1c), and glycated albumin and can be calculated from CGM data (Mohan et al., 2023). The TIR values exhibit a significant correlation with the microvascular and macrovascular complications of diabetes and are critical for quality glucose management (Yapanis et al., 2022).

Various methods, including lifestyle modifications, medications, and regular blood glucose monitoring, are used in diabetes management (Cloete, 2022). Glucagon-like peptide-1 receptor agonists (GLP-1RAs) are a class of drugs that have been gaining more attention in recent years for their efficacy and safety in treating type 2 diabetes mellitus (T2DM) (Nauck et al., 2021). GLP-1RAs improve glycemic control by stimulating insulin secretion, inhibiting glucagon release, delaying gastric emptying, and suppressing appetite (Trico and Solini, 2021). Furthermore, several clinical trials have revealed the benefits of GLP-1RA treatment in reducing body weight and improving cardiovascular outcomes in patients with T2DM (Honigberg et al., 2020; Taha et al., 2022).

Several recent randomized controlled trials (RCTs) have shown that GLP-1RAs can significantly improve TIR and reduce blood glucose fluctuations (Sofizadeh et al., 2019; Frias et al., 2023; Zhang et al., 2023). However, applying RCT results to clinical practice has certain limitations. Real-world clinical evidence supporting the effect of GLP-1RA on improving TIR is lacking. To address this, this study examined the effects of GLP-1RA-based treatment strategies on TIR among patients with T2DM in real-world clinical settings.

## 2 Materials and methods

### 2.1 Study design and patients

A multicenter, retrospective, real-world study was conducted by enrolling patients with T2DM who underwent either outpatient or inpatient treatment across five hospitals in the Guangdong Province from August 2019 to June 2023. The inclusion criteria for this study were as follows: age ≥18 years, diagnosed with T2DM, previously used a CGM system, and received GLP-1RA or oral antidiabetic drug (OAD) treatment for at least 3 months. Patients diagnosed with type 1 diabetes were excluded. The Ethics Committee of the First Affiliated Hospital of Shantou University Medical College approved the study and waived the requirement for informed consent (No. B-2023-095). This study was registered with ClinicalTrials.gov (ChiCTR2300073697). The study was conducted in compliance with the Declaration of Helsinki.

Patients were assigned to either the GLP-1RA or OAD groups based on their intake of hypoglycemic agents. The baseline data on patients were collected at the beginning of GLP-1RA treatment, including the following: age, sex, disease duration, weight, body mass index (BMI), HbA1c levels, and use of hypoglycemic agents. Additionally, the CGM data (from at least 235 h), such as TIR, tight TIR, time above range (TAR), time below range (TBR), mean glucose, standard deviation (SD), and glycemic variability, were collected.

### 2.2 Propensity score matching

Patients from the OAD group served as controls and were matched in a 1:1 ratio to their GLP-1RA counterparts using the nearest-neighbor approach, according to age, gender, diabetes duration, baseline BMI, baseline HbA1c level, baseline TIR, and the types of OADs used in both groups.

### 2.3 Outcomes

The primary endpoint was the TIR after 3–6 months of treatment. Secondary endpoints assessed at 3–6 months of treatment included tight TIR, TAR, TBR, mean glucose, SD, glycemic variability, and HbA1c, in addition to CGM data from the first month of treatment. A subgroup analysis based on GLP-1RA administration was also performed.

### 2.4 Statistical analysis

The Kolmogorov–Smirnov test was used to test the normality of continuous variables. The independent samples *t*-test, Mann–Whitney *U* test, and χ^2^ test were used to compare baseline characteristics between the two groups. The primary endpoint was then analyzed using an independent-sample *t*-test. Secondary endpoints were analyzed using the independent-sample *t*-test or Mann–Whitney *U* test. Subgroup analyses were performed using one-way ANOVA and Dunnett’s test. Statistical analysis was performed using Statistical Analysis System (SAS) version 9.4. *p*-values <0.05 (two-sided) were considered statistically significant.

## 3 Results

We identified a total of 566 patients who used the CGM system and received either GLP-1RA or OAD treatment. After propensity score matching, 101 of these patients were categorized into the GLP-1RA group, which received GLP-1RA-based treatment, including liraglutide (n = 24), semaglutide (n = 35), dulaglutide (n = 2), or polyethylene glycol loxenatide (PEG-Loxe) (n = 40). Additional 101 patients treated with OADs were matched as the control. A total of 202 patients were included in this study, with no difference in baseline characteristics between groups post-matching (Table 1).

**TABLE 1:** Baseline characteristics of patients before and after propensity score matching.

	Before propensity score matching			After propensity score matching		
	GLP-1RA	OAD	*p*-value	GLP-1RA	OAD	*p*-value
n	119	447		101	101	
Women, n (%)	63 (52.9)	178 (39.8)	0.013	54 (53.5)	52 (51.5)	0.778
Age, y	62.3 (13.6)	54.2 (12.3)	<0.0001	62.4 (13.8)	62.4 (10.9)	0.992
Duration, y	10.0 (5.0, 15.8)	3.0 (0.5, 8.1)	<0.0001	9.0 (5.0, 15.0)	9.0 (4.5, 14.0)	0.565
Body weight, kg	67.2 (12.9)	65.8 (12.0)	0.242	67.1 (12.5)	67.9 (9.3)	0.602
BMI, kg/m^2^	26.3 (1.7)	25.7 (3.4)	0.001	26.4 (1.8)	26.5 (1.4)	0.743
HbA1c, %	8.42 (1.13)	8.48 (0.97)	0.558	8.45 (1.10)	8.38 (0.96)	0.604
TIR (3.9–10.0 mmol/L), %	43.7 (15.2)	47.1 (19.1)	0.093	42.1 (13.1)	42.5 (15.6)	0.841
TAR (>10.0 mmol/L), %	55.1 (16.2)	51.8 (19.6)	0.109	56.7 (14.3)	56.4 (15.9)	0.867
TBR (<3.9 mmol/L), %	0.0 (0.0, 1.0)	0.0 (0.0, 1.0)	0.937	0.0 (0.0, 1.0)	0.0 (0.0, 1.0)	0.862
OAD use						
Biguanides, n (%)	58 (48.7)	235 (52.6)	0.457	51 (50.5)	53 (52.5)	0.778
SU/glinides, n (%)	19 (16.0)	156 (34.9)	<0.0001	12 (11.9)	14 (13.9)	0.674
AGI, n (%)	47 (39.5)	165 (36.9)	0.412	40 (39.6)	40 (39.6)	1.000
DPP-4i, n (%)	0 (0)	41 (9.2)	<0.0001	0 (0)	0 (0)	1.000
TZD, n (%)	26 (21.8)	102 (22.8)	0.822	22 (21.8)	23 (22.8)	0.866
SGLT-2i, n (%)	61 (51.3)	307 (68.7)	<0.001	53 (52.5)	56 (55.4)	0.672

AGI, alpha-glucosidase inhibitor; BMI, body mass index; DPP-4is, dipeptidyl peptidase-4 inhibitors; HbA1c, glycated hemoglobin; SGLT-2i, sodium–glucose cotransporter-2 inhibitor; SU, sulfonylurea; TAR, time above range; TBR, time below range; TIR, time in range; TZD, thiazolidinedione. Data are represented as mean (SD) or median (IQR), unless otherwise indicated.

**FIGURE 1:**
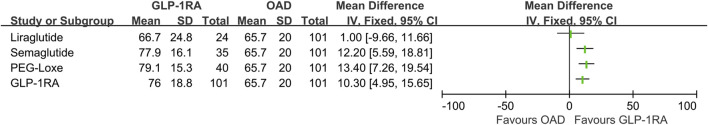
Forest plot for GLP-1RA treatment and TIR.

Following 3–6 months of treatment, the GLP-1RA group exhibited a TIR of 76.0% (SD: 18.8) compared with that of 65.7% (SD: 20.0) in the OAD group (*p* < 0.001). The tight TIR of the GLP-1RA group was significantly higher than that of the OAD group (59.0% [SD: 19.4] vs. 50.3% [SD: 19.9], *p* = 0.002), whereas TAR (18.0% [interquartile range, IQR: 6.5, 32.5] vs. 28.0% [IQR: 15.5, 45.5], *p* < 0.001) and mean glucose (8.1 mmol/L [SD: 1.7] vs. 9.0 mmol/L [1.8], *p* < 0.001) were significantly lower. FPG levels decreased by −2.3 mmol/L (1.4) in the GLP-1RA group and by −1.5 mmol/L (1.4) in the OAD group (*p* < 0.001). The GLP-1RA group exhibited a mean weight change of −2.7 kg (2.1), compared to that of −1.1 kg (1.8) in the OAD group (*p* < 0.001). No significant statistical difference was observed between the groups regarding TBR, SD, coefficient of variation (CV), and HbA1c (Figure 1; Table 2).

**TABLE 2:** Comparison of trial outcome measures between GLP-1RA and OAD, following 3–6 months of treatment.

	GLP-1RA (n = 101)	OAD (n = 101)	*p*-value
TIR (3.9–10.0 mmol/L), %	76.0 (18.8)	65.7 (20.0)	<0.001
Tight TIR (3.9–7.8 mmol/L), %	59.0 (19.4)	50.3 (19.9)	0.002
TAR (>10.0 mmol/L), %	18.0 (6.5, 32.5)	28.0 (15.5, 45.5)	<0.001
TAR (level 1: 10.1–13.9 mmol/L), %	11.0 (5.0, 19.5)	19.0 (10.5, 28.5)	<0.001
TAR (level 2: >13.9 mmol/L), %	6.0 (1.0, 12.5)	8.0 (4.0, 16.0)	0.001
TBR (<3.9 mmol/L), %	1.0 (0.0, 2.0)	0.0 (0.0, 2.5)	0.531
TBR (level 1: 3.0–3.8 mmol/L), %	1.0 (0.0, 2.0)	0.0 (0.0, 2.0)	0.587
TBR (level 2: <3.0 mmol/L), %	0.0 (0.0, 0.0)	0.0 (0.0, 0.0)	0.461
Mean glucose, mmol/L	8.1 (1.7)	9.0 (1.8)	<0.001
SD, mmol/L	1.97 (0.52)	2.05 (0.50)	0.249
CV, %	24.8 (6.7)	23.5 (6.6)	0.193
Change in HbA1c, %	−0.95 (1.06)	−0.79 (1.39)	0.424
Change in FPG, mmol/L	−2.3 (1.4)	−1.5 (1.4)	<0.001
Weight loss, kg	−2.7 (2.1)	−1.1 (1.8)	<0.001

CV, coefficient of variation; HbA1c, glycated hemoglobin; SD, standard deviation; TAR, time above range; TBR, time below range; TIR, time in range; tight TIR, tight time in range. Data are represented as mean (SD) or median (IQR), unless otherwise indicated.

In the first month of treatment, the TIR values for the GLP-1RA and OAD groups were 57.7% (SD: 21.6) and 47.1% (SD: 21.5), respectively (*p* = 0.008). The tight TIR of the GLP-1RA group was significantly higher than that of the OAD group (36.0% [IQR: 26.0, 58.0] vs. 26.5% [IQR: 21.0, 41.3], *p* = 0.003), while TAR (45.0% [IQR: 26.0, 59.0] vs. 53.5% [IQR: 40.0, 69.0], *p* = 0.011) and mean glucose (9.6 mmol/L [SD: 1.9] vs. 10.7 mmol/L [SD: 2.1], *p* = 0.003) were significantly lower. No significant statistical difference was observed between the groups regarding TBR, SD, and CV (Table 3).

**TABLE 3:** Comparison of trial outcome measures between GLP-1RA and OAD in the first month of treatment.

	GLP-1RA (n = 70)	OAD (n = 54)	*p*-value
TIR (3.9–10.0 mmol/L), %	57.7 (21.6)	47.1 (21.5)	0.008
Tight TIR (3.9–7.8 mmol/L), %	36.0 (26.0, 58.0)	26.5 (21.0, 41.3)	0.003
TAR (>10.0 mmol/L), %	45.0 (26.0, 59.0)	53.5 (40.0, 69.0)	0.011
TAR (level 1: 10.1–13.9 mmol/L), %	30.0 (15.0, 41.0)	38.0 (25.5, 46.5)	0.017
TAR (level 2: >13.9 mmol/L), %	13.0 (9.0, 17.0)	15.0 (12.0, 21.0)	0.016
TBR (<3.9 mmol/L), %	0.0 (0.0, 1.0)	0.0 (0.0, 1.3)	0.791
TBR (level 1: 3.0–3.8 mmol/L), %	0.0 (0.0, 1.0)	0.0 (0.0, 1.3)	0.706
TBR (level 2: <3.0 mmol/L), %	0.0 (0.0, 0.0)	0.0 (0.0, 0.0)	0.859
Mean glucose, mmol/L	9.6 (1.9)	10.7 (2.1)	0.003
SD, mmol/L	2.06 (0.63)	2.19 (0.69)	0.259
CV, %	21.7 (6.3)	20.7 (5.3)	0.318

CV, coefficient of variation; HbA1c, glycated hemoglobin; SD, standard deviation; TAR, time above range; TBR, time below range; TIR, time in range; tight TIR, tight time in range. Data are represented as mean (SD) or median (IQR), unless otherwise indicated.

**FIGURE 2:**
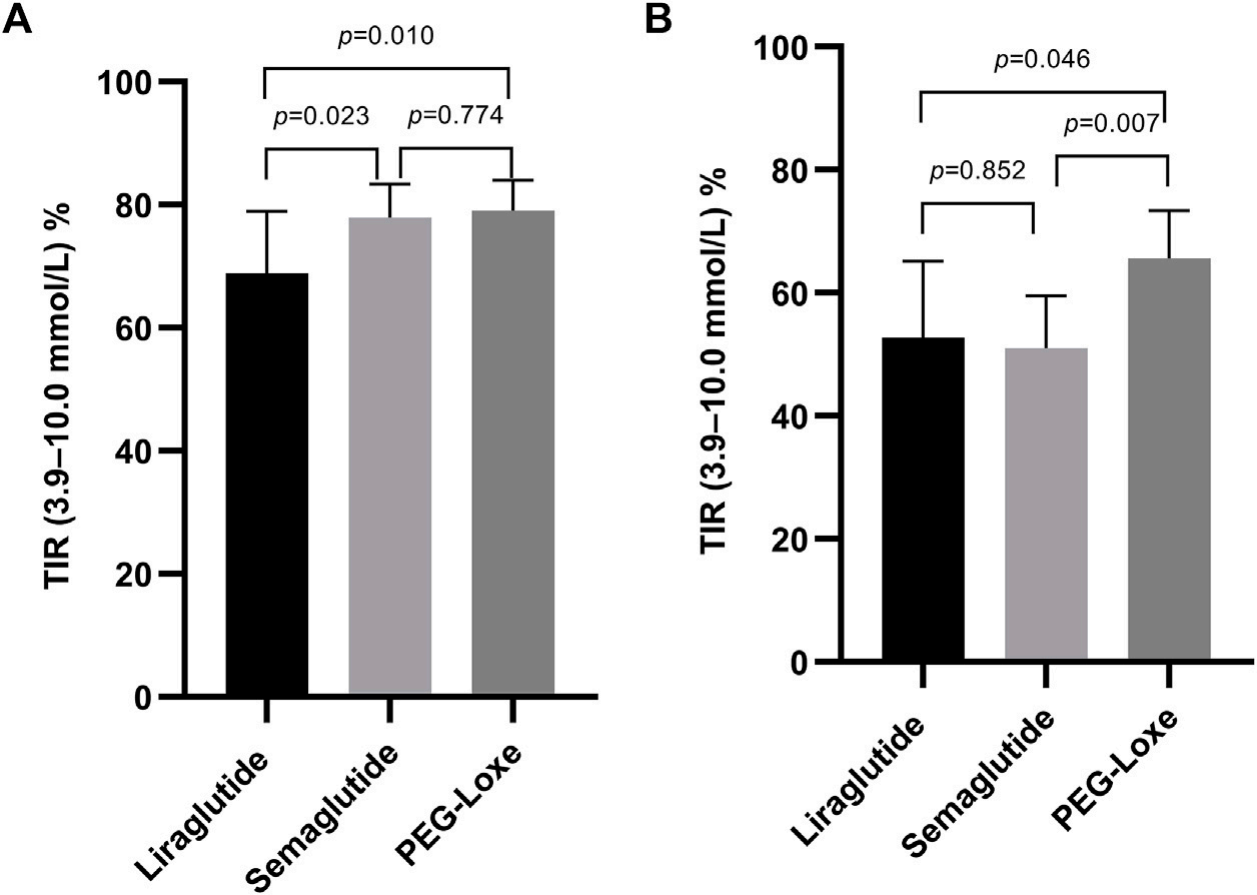
Effect on the TIR by GLP-1RA use. **(A)** TIR levels after 3–6 months of treatment. **(B)** TIR levels in the first month of treatment. Data are represented as the mean (95% CI).

Subgroup analysis indicated that after 3–6 months of treatment, PEG-Loxe and semaglutide administration significantly improved TIR compared to liraglutide administration (both *p* < 0.05) (Table 4; Figure 2A). Furthermore, in the first month of treatment, PEG-Loxe significantly improved TIR compared to both liraglutide and semaglutide (both *p* < 0.05) (Table 5; Figure 2B). Only two patients received dulaglutide treatment, so they were omitted from the subgroup analysis.

**TABLE 4:** Subgroup analysis by GLP-1RA after 3–6 months of treatment.

	Liraglutide (n = 24)	Semaglutide (n = 35)	PEG-Loxe (n = 40)	*p*-value
TIR (3.9–10.0 mmol/L), %	66.7 (56.2, 77.1)	77.9 (72.3, 83.4)	79.1 (74.2, 84.0)	0.024
Tight TIR (3.9–7.8 mmol/L), %	50.8 (41.3, 60.2)	61.3 (55.5, 67.0)	60.9 (54.9, 66.9)	0.074
TAR (>10.0 mmol/L), %	31.4 (20.5, 42.3)	20.2 (14.3, 26.0)	19.5 (14.5, 24.4)	0.037
TAR (level 1: 10.1–13.9 mmol/L), %	19.1 (12.9, 25.3)	13.1 (9.6, 16.6)	12.5 (9.4, 15.5)	0.058
TAR (level 2: >13.9 mmol/L), %	12.3 (7.2, 17.3)	7.1 (4.6, 9.6)	7.2 (4.9, 9.5)	0.043
TBR (<3.9 mmol/L), %	2.0 (0.5, 3.4)	2.0 (0.7, 3.2)	1.5 (0.9, 2.0)	0.722
TBR (level 1: 3.0–3.8 mmol/L), %	1.5 (0.5, 2.6)	1.5 (0.7, 2.4)	1.3 (0.8, 1.7)	0.821
TBR (level 2: <3.0 mmol/L), %	0.4 (0.0, 0.9)	0.4 (0.0, 0.9)	0.2 (0.0, 0.4)	0.555
Mean glucose, mmol/L	8.9 (8.0, 9.8)	7.9 (7.4, 8.4)	7.9 (7.5, 8.3)	0.046
SD, mmol/L	1.98 (1.81, 2.16)	1.93 (1.75, 2.11)	2.02 (1.83, 2.20)	0.774
CV, %	23.2 (20.6, 25.7)	24.7 (22.6, 26.7)	25.6 (23.1, 28.1)	0.377
Change in HbA1c, %	−0.83 (0.86)	−0.94 (1.35)	−0.95 (0.81)	0.927
Change in FPG, mmol/L	−2.0 (1.1)	−2.5 (1.6)	−2.5 (1.3)	0.271

CV, coefficient of variation; HbA1c, glycated hemoglobin; PEG-Loxe, polyethylene glycol loxenatide; SD, standard deviation; TAR, time above range; TBR, time below range; TIR, time in range; tight TIR, tight time in range. Data are represented as mean (SD) or median (IQR), unless otherwise indicated.

**TABLE 5:** Subgroup analysis by GLP-1RA use in the first month of treatment.

	Liraglutide (n = 13)	Semaglutide (n = 26)	PEG-Loxe (n = 31)	*p*-value
TIR (3.9–10.0 mmol/L), %	52.0 (41.0, 63.0)	50.7 (42.6, 58.8)	65.9 (57.8, 73.9)	0.015
Tight TIR (3.9–7.8 mmol/L), %	32.6 (20.7, 44.5)	36.1 (28.5, 43.6)	50.2 (41.7, 58.8)	0.013
TAR (>10.0 mmol/L), %	45.3 (31.9, 58.7)	47.8 (39.3, 56.2)	33.2 (25.0, 41.3)	0.035
TAR (level 1: 10.1–13.9 mmol/L), %	31.4 (21.5, 41.3)	32.6 (26.4, 38.8)	22.3 (16.7, 27.9)	0.034
TAR (level 2: >13.9 mmol/L), %	13.9 (9.2, 18.7)	15.2 (12.1, 18.2)	10.9 (8.1, 13.7)	0.106
TBR (<3.9 mmol/L), %	2.7 (0.0, 7.3)	1.5 (0.4, 2.6)	1.0 (0.3, 1.6)	0.404
TBR (level 1: 3.0–3.8 mmol/L), %	2.2 (0.0, 5.6)	1.3 (0.5, 2.2)	0.8 (0.3, 1.4)	0.401
TBR (level 2: <3.0 mmol/L), %	0.5 (0.0, 1.7)	0.2 (0.0, 0.4)	0.1 (0.0, 0.3)	0.421
Mean glucose, mmol/L	9.9 (8.7, 11.2)	10.1 (9.4, 10.9)	9.1 (8.4, 9.7)	0.103
SD, mmol/L	2.15 (1.73, 2.58)	2.10 (1.85, 2.36)	1.97 (1.75, 2.20)	0.620
CV, %	22.0 (18.7, 25.3)	21.4 (18.4, 24.5)	21.9 (19.8, 24.0)	0.954

CV, coefficient of variation; HbA1c, glycated hemoglobin; PEG-Loxe, polyethylene glycol loxenatide; SD, standard deviation; TAR, time above range; TBR, time below range; TIR, time in range; tight TIR, tight time in range. Data are represented as mean (SD) or median (IQR), unless otherwise indicated.

## 4 Discussion

**FIGURE 3:**
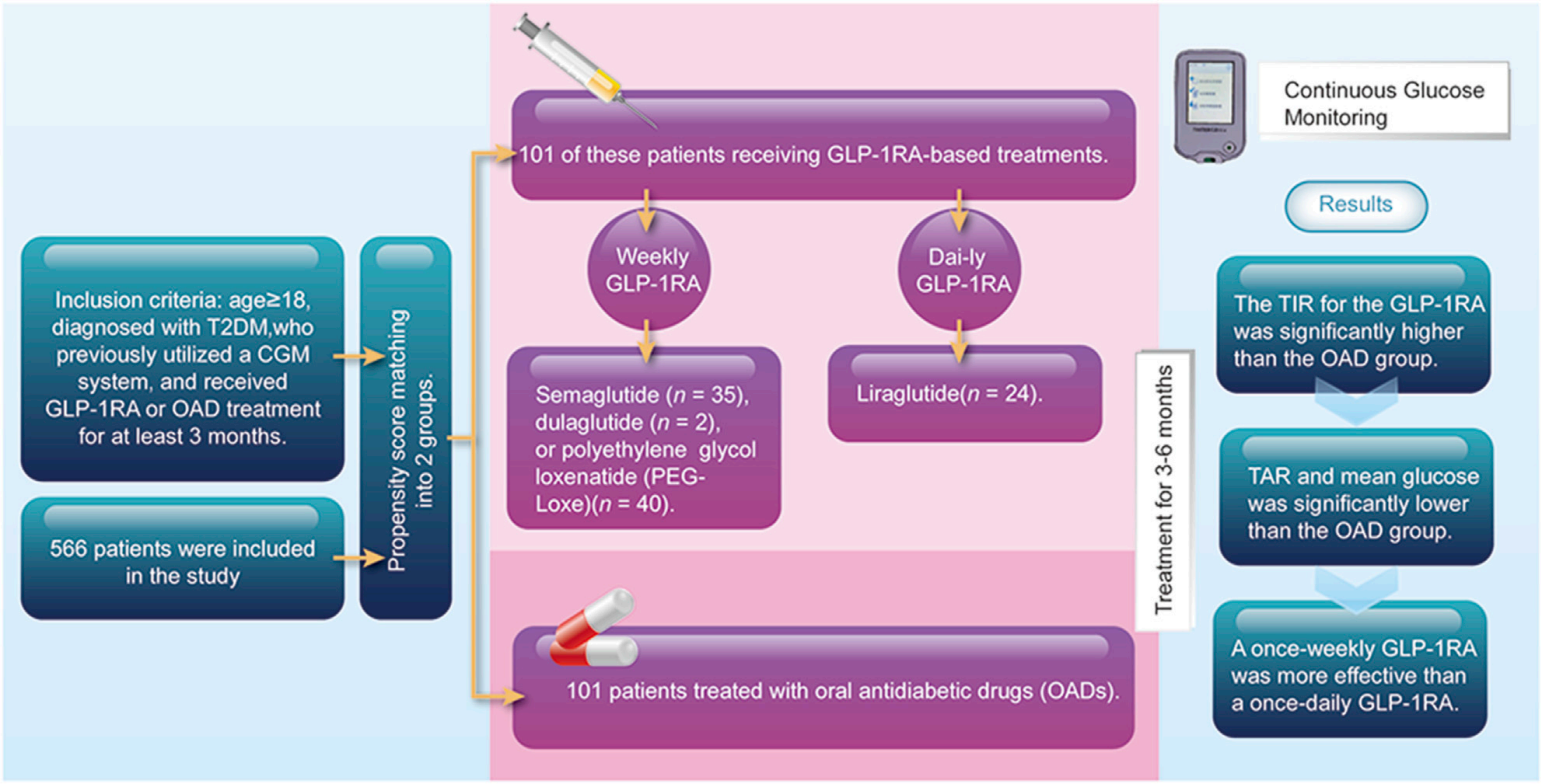
Summarized figure.

Our study is the first to conduct a retrospective comparison of the impacts of GLP-1RA-based treatment strategies and oral treatment strategies on TIR among patients with T2DM in a real-world setting. By using propensity score matching, we minimized the confounding and selection biases of covariates. Our findings suggest a significant improvement in TIR among patients with T2DM treated with GLP-1RA-based strategies for 3–6 months compared with those treated with OADs (Figure 3).

For glucose monitoring in patients with diabetes, the HbA1c level is considered the “gold standard” in clinical settings for assessing long-term glycemic control (Jendle et al., 2016). However, the HbA1c level reflects the average blood glucose level over the previous 3 months (Chen et al., 2022). According to the 2023 American Diabetes Association’s (ADA) *Standards of Care in Diabetes*, CGM continues to gain prominence in glucose monitoring, with TIR as an alternative indicator of HbA1c (ElSayed et al., 2023). Higher TIR was associated with a reduced risk of chronic kidney disease, diabetic retinopathy, major cardiovascular adverse events, and all-cause death (Yapanis et al., 2022). These findings support its important role in clinical practice. In several published RCT studies, TIR reached 66.7% after 6 months of liraglutide treatment (Sofizadeh et al., 2019), 76.2% after 8 months of semaglutide treatment (Frias et al., 2023), and 83.1% after 6 months of dulaglutide treatment (Jendle et al., 2016). Our prior study observed a TIR of 81.4% after 6 months of PEG-Loxe treatment (Zhang et al., 2023). The findings of this study were similar, with a TIR of 76.0% after 3–6 months of GLP-1RA-based treatment, exceeding the 70% recommended by the international consensus (Battelino et al., 2019).

In a previous study, TIR was negatively correlated with HbA1c; every 10% increase in TIR was associated with a 0.3% decrease in HbA1c (Shah et al., 2021). In this study, TIR in the GLP-1RA group increased from 42.1% to 76.0% and that in the OAD group increased from 42.5% to 65.7%. According to the quantitative relationship mentioned above, the corresponding reduction of HbA1c in the GLP-1RA and OAD groups should be 1.02% and 0.70%, respectively, which is consistent with the actual reduction of HbA1c in the two groups (0.95% and 0.79%).

TAR primarily reflects postprandial blood glucose control status (Babaya et al., 2021). The hypoglycemic effect of GLP-1RAs is achieved through several mechanisms, including promoting insulin secretion, inhibiting glucagon secretion, slowing gastric emptying, and inhibiting central appetite (Zhao et al., 2021). The present study revealed that the TAR after 3–6 months of GLP-1RA treatment was 18.0%, falling below the 25% recommended by the international consensus (Battelino et al., 2019).

GLP-1RA inhibits appetite, increases satiety, restricts caloric intake, and reduces the body weight through central mechanisms (Ard et al., 2021). The first is primarily exhibited by acting on the circumventricular organs outside the blood–brain barrier. These circumventricular organs that lack a functional blood–brain barrier, such as the area postrema and hypothalamic arcuate nucleus, have been shown to be targets for the systemic administration of GLP-1RA. Second, GLP-1RA activates vagal afferent neurons to transmit signals to the nucleus tractus solitarius (McLean et al., 2021). GLP-1RA administration causes an average weight loss of 2–5 kg in patients with T2DM (Laurindo et al., 2022). In this study, the weight loss in the GLP-1RA group was 2.7 kg, which is consistent with previous studies (Laurindo et al., 2022). This suggests that GLP-1RA is effective as a weight loss agent in patients who are overweight in real-world settings.

Long-acting GLP-1RAs offer less variation in plasma concentrations, thereby suggesting a stable hypoglycemic effect (Gentilella et al., 2019). Pharmacokinetic data on liraglutide, semaglutide, and PEG-Loxe show that semaglutide and PEG-Loxe maintain more stable plasma concentrations upon reaching a steady state (Watson et al., 2010; Yang et al., 2015; Overgaard et al., 2019). Subgroup analysis indicated a TIR of 66.7% after 3–6 months of liraglutide treatment, aligning with prior research findings (Sofizadeh et al., 2019). Compared with liraglutide, semaglutide and PEG-Loxe significantly improved TIR after 3–6 months of treatment. This suggests that the improvements in TIR from weekly GLP-1RA administration may be superior to those from daily GLP-1RA administration.

The initial recommended dose of semaglutide is 0.25 mg; however, the package inserts for semaglutide state that the 0.25-mg dose is not effective for glycemic control (Novo Nordisk, 2017). A previous cohort study found that the TIR after 3 months of semaglutide treatment was 50.8% (Al Hayek and Al Dawish, 2022). The current study observed similar results, with a TIR of 51.0% after 1 month of semaglutide treatment, which was significantly lower than that found with PEG-Loxe treatment. This suggests the need to focus on glycemic management when starting treatment with semaglutide at 0.25 mg.

In this study, patients in the GLP-1RA group also received oral medication. The most commonly used agents were sodium–glucose cotransporter-2 inhibitors, metformin, alpha-glucosidase inhibitors, and thiazolidinediones. Due to the retrospective study design, we were not able to collect information on changes in medication dosing, and it is possible that multiple patients deviated from the prescribed dosing regimen. In addition, lifestyle intervention runs throughout the treatment of diabetes; however, due to the retrospective design of the study, data on lifestyle interventions could not be obtained. Therefore, the results should be interpreted with caution.

This study has some limitations. First, the sample size was relatively small and was not big enough to draw statistically valid conclusions, especially for dulaglutide. There were only two patients on dulaglutide in this study, and the data on dulaglutide were not included in the subgroup analysis. Second, due to the retrospective study design, despite the presence of a control group, it is not possible to completely exclude differences in lifestyle interventions and the influence of dose changes in other hypoglycemic drugs in this real-world study.

In conclusion, we found that GLP-1RA-based treatment strategies could be superior to oral treatment strategies for improving TIR in patients with T2DM in a real-world setting. Moreover, weekly GLP-1RA administration may be more effective than daily GLP-1RA administration. As the use of GLP-1RAs increases, future long-term research is needed to confirm these findings.

## Data Availability

The original contributions presented in the study are included in the article/Supplementary Material; further inquiries can be directed to the corresponding author.
